# Copula modeling of gene coexpression in single-cell RNA sequencing data

**DOI:** 10.64898/2025.12.04.692380

**Published:** 2025-12-09

**Authors:** Connor Puritz, Rosemary Braun

**Affiliations:** 1 Engineering Sciences and Applied Mathematics, McCormick School of Engineering, Northwestern University, Evanston, IL 60208, USA; 2 Department of Molecular Biosciences, Weinberg College of Arts and Sciences, Northwestern University, Evanston, IL 60208, USA; 3 NSF-Simons National Institute for Theory and Mathematics in Biology, Chicago, IL 60611, USA; 4 Santa Fe Institute, Santa Fe, NM 87501, USA

## Abstract

Single-cell RNA sequencing (scRNA-seq) has become an indispensable tool for studying biological systems at the cellular level. It has therefore has become increasingly important to develop accurate statistical models of scRNA-seq data. While many models have been proposed to characterize transcript expression of individual genes, comparatively little attention has been paid to modeling gene coexpression. Copula modeling offers a flexible approach to modeling gene coexpression by linking models of individual genes together using copula functions. Despite the growing popularity of copula models, their utility for modeling scRNA-seq data has not been thoroughly explored. Here we evaluated six copula models on their ability to model gene coexpression in scRNA-seq data. Using a diverse collection of reference datasets, we evaluated each copula model’s accuracy and efficiency in reproducing gene coexpression patterns. Our results show that Gaussian copulas provide the best balance between accuracy and speed, with more flexible but expensive copula models providing only a marginal improvement in accuracy while requiring a much longer time to fit. Vine copulas show promise in being able to achieve high accuracy, but current implementations are unable to scale to the large size of typical scRNA-seq datasets.

## 1 Introduction

Since it was first developed, single-cell RNA sequencing (scRNA-seq) has become a popular tool for analyzing biological systems on the cellular level by allowing quantification of the transcriptome of individual cells. Compared to other technologies for transcriptome analysis, scRNA-seq presents a number of unique challenges. The number of cells profiled is often in the thousands to millions, several orders of magnitude larger than the sample sizes of bulk RNA-seq or microarray experiments. Furthermore, the transcript counts are much sparser, with expression observed for only a small percentage of genes in each cell (Zappia et al., 2017), and the data may also suffer from zero-inflation. This has led to the development of a plethora of methods designed specifically to address these characteristics of scRNA-seq data.

A common starting point for many scRNA-seq methods is the construction of a statistical model of transcript counts in individual cells. It is important to not only model the expression of individual genes but also the coexpression of genes, as analysis of gene coexpression can provide insight to many important biological processes (e.g. Stuart et al., 2003; Ho et al., 2007; Braun et al., 2008; Gaiteri et al., 2014; Ruprecht et al., 2017; Panwar et al., 2021; Wang et al., 2021). However, many of the statistical models of scRNA-seq data that have been proposed either focus on modeling transcript counts for individual genes (e.g. Zappia et al., 2017; Li and Li, 2019) or do not explicitly model coexpression (e.g. Risso et al., 2018). The latter is important if one wishes to perform in-silico perturbations with a ground truth.

One approach for explicitly modeling coexpression is copula modeling. First introduced by Sklar (1959), copulas can be formally defined as multivariate cumulative distribution functions with uniform margins on the unit interval (Genest et al., 2024). Their utility arises from Sklar’s theorem, which states that any multivariate distribution function can be expressed in terms of its marginal distribution functions and a copula function (Sklar, 1959; Genest et al., 2024). Copulas thus link one-dimensional distributions together to yield multivariate distributions (Nelsen, 2006). Copulas provide an approach for modeling and analyzing dependencies in high-dimensional datasets, and can also be used to construct flexible high-dimensional distributions with a specified dependence structure.

In recent years, copulas have become a popular tool in omics research for modeling dependencies between genes. Zhang (2017) implemented a Bayes classifier of bulk RNA-seq data using a Gaussian copula model of transcript counts. Zhang and Shi (2017) constructed a Gaussian mixture copula model for inferring a Bayesian network from multimodal genomics data. Ray et al. (2020) proposed a copula-based statistical test for detecting differential coexpression of genes from bulk RNA-seq data. Sarkar et al. (2024) proposed a method for inferring cell-cell interactions from spatial transcriptomics data using a Gaussian copula to model joint ligand-receptor expression. ESCO (Tian et al., 2021), SPsimSeq (Assefa et al., 2020), and scDesign2 (Sun et al., 2021) are all methods for generating synthetic scRNA-seq data which use a Gaussian copula model. scDesign3 (Song et al., 2024), which extends scDesign2, generates synthetic single-cell and spatial omics data using both Gaussian and vine copula models.

Despite the extensive use of copulas in omics methods, no systematic evaluation has been performed to assess different copula models on their utility and accuracy in modeling gene dependencies. Instead, the choice of copula model has often been guided by convenience or simplicity. Here, we sought to fill this gap by evaluating six copula models on their performance in modeling gene coexpression patterns in scRNA-seq data. We fit copula models to 38 scRNA-seq datasets covering a range of cell types, tissues, organisms, and sequencing protocols. We then compared models based on their accuracy on several tasks, as well as their scalability. Finally, we give users recommendations for choosing copula models based on the priorities of their analyses.

## 2 Materials and Methods

### 2.1 Copula modeling of scRNA-seq data

Suppose that we have a scRNA-seq dataset with transcript counts for m genes from n cells. Let $X=\left(X_{1},\ldots ,X_{m}\right)$ be a random variable representing the unnormalized transcript counts across all m genes for a single cell. The support of each $X_{i}$ is the nonnegative integers. If there are no covariates to model (e.g. multiple cells types, multiple donors, etc.), we can equate the m×n count matrix with a set of n independent and identically distributed (iid) samples of X.^1^ Given a parametric family of distribution functions $\left\{H_{\theta }|\theta \in \Theta \right\}$, our goal is to learn from the dataset the parameter θ such that $H_{\theta }$ best approximates the true distribution function H of X.

According to Sklar’s theorem (Sklar, 1959), there exists a function $C:[0,1{]}^{m}\to [0,1]$ called a copula such that the distribution function of X can be written as

(1) $$H\left(x_{1},\ldots ,x_{m}\right)=C\left(F_{1}\left(x_{1}\right),\ldots ,F_{m}\left(x_{m}\right)\right),$$

where $F_{i}$ is the distribution function of the ith margin (i.e. the transcript counts for a single gene). Learning H is thus equivalent to learning the marginal distributions and the associated copula. Numerous parametric models for transcript counts of individual genes in scRNA-seq datasets have been proposed (e.g. Zappia et al., 2017; Risso et al., 2018). However, as our goal is to evaluate the copula estimators, we chose to model the marginal distributions empirically, since misspecification of the marginal models could degrade the quality of the copula estimators (see Section S1).

**Figure 1:**
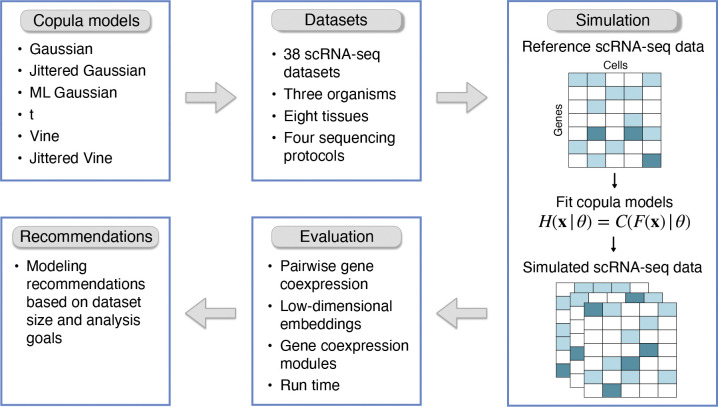
Schematic of the workflow used to evaluate copulas for modeling gene coexpression in single-cell RNA sequencing data.

In the absence of a ground truth model for a given dataset, it is difficult to evaluate if one copula model is better than another by only examining the copula functions. Instead, we reasoned that if a distribution function $H_{\theta }$ is closer to the true distribution function than another distribution function $H_{\theta^{\prime }}$, then samples drawn from $H_{\theta }$ should be harder to statistically distinguish from the reference dataset than samples drawn from $H_{\theta^{\prime }}$. We thus constructed a diverse compendium of scRNA-seq datasets. For each dataset, we fit a variety of copula models, sampled synthetic datasets from each model, and compared the samples with the reference dataset using a variety of metrics (Figure 1). Below, we describe each of these steps in detail.

### 2.2 Copula families

Here we describe the various families of copulas that we evaluated.

#### 2.2.1 Gaussian copula

The Gaussian copula is the copula of the multivariate Gaussian distribution. It is defined as

(2) $$C_{R}\left(u_{1},\ldots ,u_{d}\right)=\Phi_{R}\left(\Phi^{-1}\left(u_{1}\right),\ldots ,\Phi^{-1}\left(u_{d}\right)\right),$$

where R is a d×d correlation matrix, $\Phi^{-1}$ is the standard normal quantile function, and $\Phi_{R}$ is the distribution function of a d-dimensional Gaussian random variable with mean 0 and covariance matrix R. The Gaussian copula is one of the most commonly used copulas as it can be easily constructed in arbitrary dimensions and it is intuitively parametrized by a correlation matrix. Gaussian copulas have been previously used for modeling scRNA-seq data (Zhang, 2017; Assefa et al., 2020; Tian et al., 2021; Sun et al., 2021; Song et al., 2024; Sarkar et al., 2024). We consider three different estimators for the correlation matrix: a sample correlation matrix computed using transcript counts (see Section S3.1), a sample correlation matrix computed using jittered margins (see Section 2.2.4, Section S2), and the maximum likelihood estimator of the correlation matrix (see Section S3.2). We refer to these as Gaussian, jittered Gaussian, and ML Gaussian copulas, respectively.

#### 2.2.2 t copula

A common criticism of the Gaussian copula is that it does not adequately model tail dependence, i.e., the co-occurrence of extreme events in the tails of marginal distributions (Zeevi and Mashal, 2002). An extension of the Gaussian copula which does model tail dependence is the t copula, which is the copula of the multivariate t distribution. Similar to the Gaussian copula, the t copula is implicitly defined as

(3) $$C_{\nu ,R}\left(u_{1},\ldots ,u_{d}\right)=t_{\nu ,R}\left(t_{\nu }^{-1}\left(u_{1}\right),\ldots ,t_{\nu }^{-1}\left(u_{d}\right)\right),$$

where ν is the degrees of freedom; R is a correlation matrix; $t_{\nu }^{-1}$ is the quantile function a univariate t distribution with degrees of freedom ν; and $t_{\nu ,R}$ is the distribution function of a d-dimensional t random variable with mean 0, degrees of freedom ν, and scale matrix R. Note that the t copula converges uniformly to the Gaussian copula in the limit ν→∞ for fixed R (Zeevi and Mashal, 2002). To the best of our knowledge, the t copula has not previously been applied to omics data. We fit t copulas to transcript counts using a two-stage maximum likelihood approach (see Section S3.3).

#### 2.2.3 Vine copulas

Many parametric families of bivariate copulas have been described, each capturing a wide range of dependence structures. While most can be generalized to higher dimensions, these generalizations are often quite inflexible (Czado and Nagler, 2022). Pair-copula decompositions address this by decomposing high-dimensional copulas in terms of bivariate, or pair, copulas. The most popular pair-copula decomposition is the vine copula decomposition, which exploits the fact that any multivariate density function can be decomposed into a product of bivariate conditional density functions. To illustrate this, we consider the following example from Czado and Nagler (2022). Suppose X is a three-dimensional random variable (e.g., a trio of coexpressed genes) with joint distribution function F, marginal distribution functions $F_{i}(i=1,2,3)$, and copula C. Denote the corresponding density functions as $f,\;f_{i}$, and c, respectively. By Bayes’ theorem, the joint density function can be decomposed as

(4) $$f\left(x_{1},x_{2},x_{3}\right)=f_{3|12}\left(x_{3}|x_{1},x_{2}\right)f_{2|1}\left(x_{2}|x_{1}\right)f_{1}\left(x_{1}\right),$$

where $f_{j|D}$ denotes the conditional density function of $X_{j}$ given $\left\{X_{k}=x_{k}|k\in D\right\}$ for nonempty D⊆{1,2,3}. Note that this decomposition is not unique, and different orders of decomposition will give different copulas. We can rewrite Equation 4 in terms of pair copula densities as

(5) $$f\left(x_{1},x_{2},x_{3}\right)=c_{13;2}\left(F_{1|2}\left(x_{1}|x_{2}\right),F_{3|2}\left(x_{3}|x_{2}\right);x_{2}\right)\times \;\;c_{23}\left(F_{2}\left(x_{2}\right),F_{3}\left(x_{3}\right)\right)c_{12}\left(F_{1}\left(x_{1}\right),F_{2}\left(x_{2}\right)\right)\times \;\;f_{3}\left(x_{3}\right)f_{2}\left(x_{2}\right)f_{1}\left(x_{1}\right)$$

where $c_{13;2}$ denotes the copula density function of the conditional distribution of ($X_{1},X_{3}$) given $X_{2}=x_{2}$. In practice, the dependence on $x_{2}$ in $c_{13;2}$ is dropped to facilitate estimation, thus making vine copulas a construction rather than a decomposition (Czado and Nagler, 2022). The construction process involves selecting an order of decomposition, selecting a parametric family for each pair copula, and estimating the parameters for each pair copula. Vine copulas have been previously used to model scRNA-seq data (Fuetterer et al., 2019; Song et al., 2024). We model vine copulas using rvinecopulib (Nagler and Vatter, 2023) and evaluated vine copulas fit directly to transcript count data as well as vine copulas fit using jittered margins (see Section 2.2.4, Section S2, and Section S3.4).

#### 2.2.4 Jittered copulas

There are two possible concerns with copula modeling of discrete data. The first is that the copula of a discontinuous distribution is not identifiable. Sklar’s theorem (Sklar, 1959) states that if the margins of the joint distribution are all continuous, then there exists a unique copula satisfying Equation 1. However, if at least one margin is discontinuous, then there exist infinitely many copulas that satisfy Equation 1 (Nasri and Remillard, 2023). Nevertheless, while the copula may not be identifiable, copula parameters within a fixed copula family often are, and thus parameter estimation is not inherently problematic (see Nasri and Remillard (2023) for a more detailed discussion). The second more practical concern is that parameter estimation is generally more computationally expensive for discrete data than continuous data, and especially so for count data (Panagiotelis et al., 2012). One approach to address these concerns is to “jitter” discrete distributions through the introduction of uniform random noise to make the margins continuous (see Section S2). Sun et al. (2021) used jittered Gaussian copulas for scRNA-seq modeling, while Song et al. (2024) used both jittered Gaussian and jittered vine copulas.

#### 2.2.5 Independence copula

As a baseline for comparison, we also evaluated the independence copula, which is defined as

(6) $$C\left(u_{1},\ldots ,u_{d}\right)=\prod_{i=1}^{d}u_{d}.$$

If a distribution’s copula is the independence copula, then the distribution’s margins are independent. When used to model scRNA-seq data, this would imply the expression of each gene has no dependence on the expression of any other gene.

### 2.3 Datasets

Ten publicly-available scRNA-seq studies were selected to construct reference datasets (Table 1). Eight of the studies sequenced human (*Homo sapiens*) cells from a total of seven different tissues. The other two studies sequenced mouse (*Mus musculus*) cells and pig (*Sus scrofa domesticus*) cells. The studies used either a 10× 3’ assay, a 10× 5’ assay, or both. Each study was broken down into several datasets, with each dataset serving as a single reference dataset for simulation. A total of 38 reference datasets were constructed. To minimize covariates that would require more complex modeling, and to ensure that the iid assumptions were met, all datasets were chosen to be from a single donor/sample, of a single cell type, and generated using the same sequencing protocol. In each dataset, genes with zero expression across all cells were removed and counts were log-normalized.

The genes selected for modeling in each dataset varied between the different evaluations. For the evaluation of pairwise coexpression and low-dimensional representations, the same sets of genes were used. Genes selected for modeling were either top highly variable genes (HVGs) or genes from a predefined gene set. Some datasets had both HVGs and gene sets modeled, resulting in a total of 48 datasets being used for these evaluations. The number of genes selected in each case was between 10 and 50. HVGs were identified using the scran package (Lun et al., 2016). Gene sets were downloaded from the Kyoto Encyclopedia of Genes and Genomes (KEGG) (Kanehisa and Goto, 2000). The gene sets selected were breast cancer (hsa05224), estrogen signaling pathway (hsa04915), cardiac muscle contraction (hsa04260), and insulin secretion (hsa04911). These correspond with the tissues sequenced by Reed et al. (2024), Jones et al. (2024), Litviňuková et al. (2020), and Tritschler et al. (2022), respectively. Three of the gene sets contained more than 50 genes and were downsampled. To do so, we first constructed undirected gene-gene graphs for each gene set using graphite (Sales et al., 2012). Leiden clustering was then performed on each graph, and a cluster with 10–50 genes was chosen, yielding a “core” network. For a given dataset and gene set, genes in the gene set that were expressed by less than 2% of cells were removed.

A minimum of 500 genes is recommended for gene coexpression module identification (Morabito et al., 2023). As such, for the evaluation of gene coexpression module preservation, we needed to increase the number of genes modeled in each dataset. For each of the 38 datasets, we removed genes expressed in less than 5% of cells. We then identified the top HVGs in each dataset, and kept between 500 and 1000 genes per dataset. The distribution of cells and gene counts across reference datasets is shown in Figure S1. A description of each dataset is given in Table S1.

### 2.4 Model evaluation

Each reference scRNA-seq dataset was first split into equal sized training and testing datasets. The same set of train-test splits was used for all copula families for a given evaluation. For the evaluation of pairwise coexpression and low-dimensional representations, 50 train-test splits were used. For the evaluation of gene coexpression networks, only 10 train-test splits were used due to the longer run time of the evaluation.

Joint distribution functions were fit to each of the training datasets by first fitting marginal models (Section S1) and then copula models (Section S3). To account for sampling variability, each joint distribution was sampled 20 times to generate 20 synthetic datasets. The statistics for each of the evaluations were first averaged over the 20 synthetic datasets per train-test split, and then averaged again over all train-test splits, resulting in one statistic per copula per reference dataset. The different evaluations we performed are described below.

#### 2.4.1 Pairwise coexpression

To evaluate the ability of a copula model to capture pairwise gene coexpression patterns present in a reference dataset, we computed gene-gene association matrices using six measures of pairwise association: Pearson correlation, Spearman rank correlation, Kendall rank correlation, mutual information, biweight midcorrelation, and distance correlation. Given a synthetic dataset and a reference dataset, we computed the respective pairwise similarity matrices $M_{s}$ and $M_{r}$, and then computed the Frobenius norm of their difference:

(7) $$Err\left(M_{s},M_{r}\right)={\left\|M_{s}-M_{r}\right\|}_{F}=\sqrt{\sum_{i=1}^{m}\;\sum_{j=1}^{m}\;{\left|{\left(M_{s}\right)}_{ij}-{\left(M_{r}\right)}_{ij}\right|}^{2}}$$

We refer to this quantity as Frobenius error.

#### 2.4.2 Low-dimensional representations

Next, we sought to evaluate how similar synthetic datasets looked to reference datasets when embedded in a low-dimensional space. Differences between the embeddings could be driven by differences in either pairwise or higher order coexpression patterns. For each reference dataset, we performed principal component analysis (PCA) and projected the dataset onto its first two principal components (PCs). We then projected each corresponding synthetic dataset onto this PC space and tested whether the differences between the embeddings were statistically distinguishable. We did so using the Fasano-Franceschini test (Fasano and Franceschini, 1987), which is a multivariate extension of the Kolmogorov-Smirnov test. The Fasano-Franceschini test has been shown to have good power for two-sample multivariate goodness-of-fit testing, especially when testing copula alternatives (Puritz et al., 2023). The null hypothesis of the two-sample Fasano-Franceschini test is that the two samples being tested were drawn from the same distribution. Since the marginal distributions for a given reference dataset are fixed, any difference in the embeddings is due to the choice of copula model. As such, we would expect larger (less significant) p-values for better fitting copula models.

#### 2.4.3 Gene coexpression modules

We also sought to evaluate how copula models can impact gene coexpression module identification. We reasoned that since coexpression modules are determined by analyzing gene-gene correlations, a more accurate copula model would lead to better preservation of coexpression modules in synthetic datasets. We identified gene coexpression modules using hdWGCNA (Morabito et al., 2023), which is an extension of WGCNA (Langfelder and Horvath, 2008) for scRNA-seq data.

Metacells were constructed in training and testing datasets using the MetacellsByGroups function, with the target number of metacells set to 500. We then identified gene coexpression modules in the testing datasets using the TestSoftPowers and ConstructNetwork functions. Next, we projected the modules from each testing dataset onto the corresponding simulated datasets using the ProjectModules function. We then performed module preservation testing using the ModulePreservation function with 100 permutations. We quantified module preservation using the $Z_{\text{summary}}$ statistic, which is a composite statistic that combines several Z statistics which individually measure different aspects of module preservation. Simulations have shown this statistic to perform well at distinguishing preserved from non-preserved modules (Langfelder et al., 2011). Since the number of modules identified varied between reference datasets, we computed the mean $Z_{\text{summary}}$ statistic across all modules, and used this value to compare copula models. We denote this value as ${\overset{‾}{Z}}_{\text{summary}}$.

#### 2.4.4 Run time

To evaluate the run time for fitting different copulas, we randomly downsampled the Bailey24 dataset (Bailey et al., 2024) to generate datasets with 100, 1000, 2000, 3000, 4000, or 5000 cells and 10, 20, 30, 40, or 50 genes. For each combination of the number of cells and genes, we fit Gaussian and jittered Gaussian copulas on 100 random datasets. Vine, jittered vine, ML Gaussian, and t copulas were fit only on three random datasets each due to their longer run times. The reported times for each copula represent the average across all datasets. Each task was run on a single CPU core of an Intel Xeon CPU (Gold 6230 @ 2.10GHz, Gold 6338 @ 2.0GHz, or Platinum 8592+ @ 1.9GHz) and was provided with a maximum of 50GB of RAM. Fitting was terminated if the wall time exceeded 10 days.

## 3 Results

### 3.1 Preservation of pairwise gene coexpression

**Figure 2:**
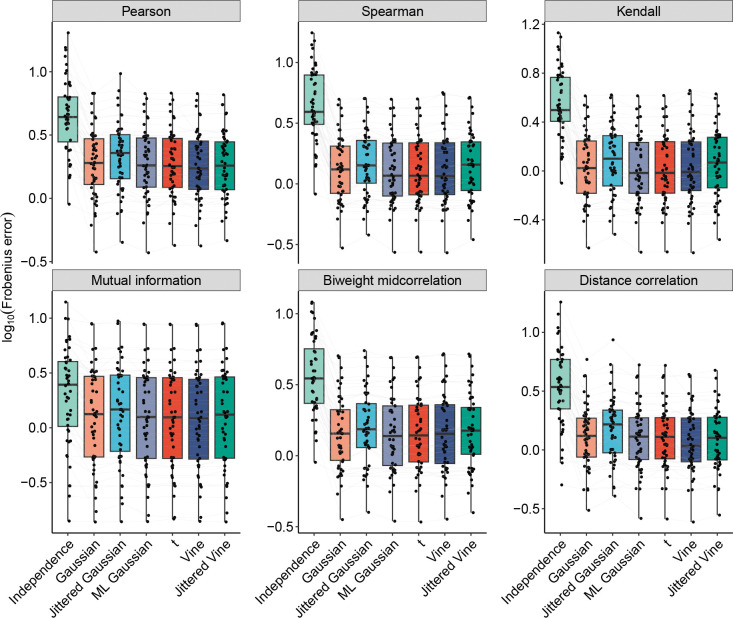
Evaluation of copula models on preserving pairwise gene associations. Comparison of copula models on their ability to preserve pairwise gene associations in reference datasets. Each point represents the average Frobenius error between gene-gene association matrices of synthetic datasets and the corresponding reference dataset. Measures of association include Pearson correlation, Spearman rank correlation, Kendall rank correlation, mutual information, biweight midcorrelation, and distance correlation. Different models of the same reference dataset are connected by a line.

We first evaluated the different copula models on their ability to accurately capture pairwise gene coexpression patterns present in reference datasets (see Section 2.4.1). Coexpression was quantified using six different commonly used measures of association, and copulas were evaluated on the Frobenius error between the pairwise gene association matrices of each synthetic dataset and corresponding reference dataset (Figure 2).

For each measure of association, we tested whether the error was significantly different (paired Wilcoxon rank-sum test with FDR correction, α<0.05) between each pair of copula families (excluding independence copulas). Out of the 90 pairwise comparisons, 51 were significant (see Table S2). However, many of these comparisons had extremely small effect sizes, suggesting that the differences between models may be negligible. Nevertheless, some patterns are notable: out of the 26 comparisons with an effect size larger than 0*.*10, 20 comparisons involve jittered Gaussian copulas, with jittered Gaussian copulas having a higher error than the other family in every comparison. Furthermore, in none of those 26 comparisons do vine, ML Gaussian, or t copulas perform worse than another family. We also observe that there is generally an increase in error when using jittered copulas as opposed to fitting the same type of copula (Gaussian or vine) directly to transcript counts. ML Gaussian and t copulas have nearly identical performance with each other and similar performance to vine copulas across all measures of association.

### 3.2 Similarity of low-dimensional embeddings

To further assess model accuracy, we evaluated how similar embeddings of synthetic datasets were to reference datasets in a low-dimensional space (see Section 2.4.2). Since the marginal distributions remain fixed for each reference dataset, any differences in embeddings are due solely to the choice of copula. These could be driven by differences in either pairwise or higher order gene coexpression patterns. For each pair of synthetic and reference datasets, we projected both onto the two-dimensional principal component space of the reference dataset. The similarity of the two embeddings was then evaluated using the Fasano-Franceschini test, which evaluates the null hypothesis that two samples were drawn from the same underlying distribution. Larger p-values therefore indicate that the embeddings are harder to statistically distinguish, suggesting that the copula model more accurately captures coexpression patterns in the reference dataset.

**Figure 3:**
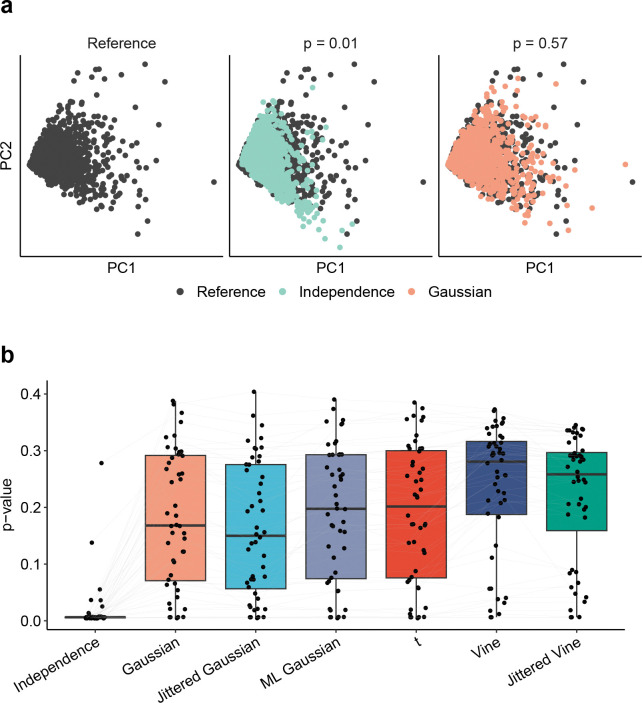
Evaluation of copula models using principal component embeddings. (a) Example of synthetic datasets generated from two different copula models of the same reference dataset embedded in the two-dimensional principal component (PC) space of the reference dataset. The p-value for the Fasano-Franceschini test between the embeddings is shown above each plot. (b) Evaluation of PC embeddings of synthetic datasets generated from different copula models. Each point represents the p-value from the Fasano-Franceschini test comparing the two-dimensional embeddings of a synthetic dataset and the corresponding reference dataset in the PC space of the reference dataset. A larger p-value indicates that the two embeddings are harder to statistically distinguish. Different models of the same reference dataset are connected by a line.

As before, jittered Gaussian copulas perform worst, tending to have the smallest (worst) p-values besides the independence copula (Figure 3). Vine and jittered vine copulas perform best, with vine copulas generally having slightly larger (better) p-values that jittered vine copulas on the same reference datasets. Gaussian, ML Gaussian, and t copulas all perform similarly. These results are consistent when the number of principal components used is increased Figure S2).

### 3.3 Gene coexpression network preservation

In the previous two evaluations, we examined the similarity of synthetic and reference count matrices. Next, we sought to evaluate copula models on a downstream task. A common step in the analysis of RNA-seq data is gene coexpression network analysis. In the typical methodology, networks are constructed with nodes representing genes, and the edges connecting the nodes being weighted by some measure of pairwise gene correlation. A clustering algorithm is then applied to identify groups of strongly coexpressed genes called coexpression modules (Lemoine et al., 2021). Since the choice of copula determines the coexpression patterns in synthetic datasets generated from a copula model, we reasoned that the choice of copula would impact the similarity of coexpression modules between reference and synthetic datasets.

**Figure 4:**
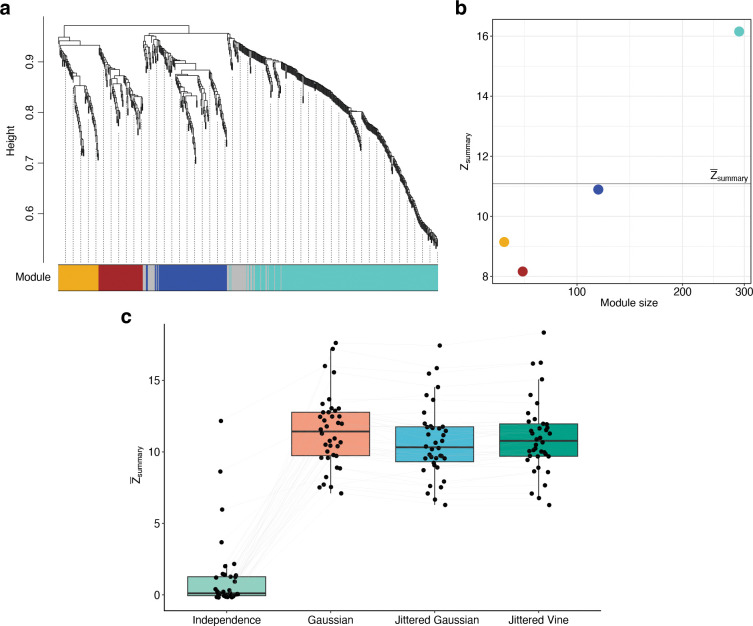
Influence of copulas on gene coexpression module preservation. (a) hdWGCNA cluster dendrogram and module assignment for genes in a reference dataset of human CD8^+^ T-cells. Module assignments are labeled by color underneath the dendrogram. Genes unassigned to a module are labeled in gray. (b) The module preservation statistic $Z_{\text{summary}}$ for the four modules identified in (a), plotted as a function of module size. The x-axis is on a logarithmic scale. The statistic ${\overset{‾}{Z}}_{\text{summary}}$ is indicated by a gray line. (c) Preservation of gene coexpression modules between reference datasets and synthetic datasets generated using different copula models. Each point represents the ${\overset{‾}{Z}}_{\text{summary}}$ statistic for a single synthetic dataset. Different models of the same reference dataset are connected by a line.

We performed gene coexpression network analysis using hdWGCNA (Morabito et al., 2023), which is an extension of the popular tool WGCNA (Langfelder and Horvath, 2008) designed specifically for scRNA-seq data (Figure 4a). Preservation of coexpression modules was quantified using the statistic $Z_{\text{summary}}$, which is a composite statistic evaluating various aspects of module preservation. The higher the value of $Z_{\text{summary}}$, the better coexpression modules were preserved. Since the number of modules identified in reference datasets varied, we compared copulas by averaging $Z_{\text{summary}}$ across all modules in each reference dataset (Figure 4b). We denote this value as ${\overset{‾}{Z}}_{\text{summary}}$.

As a minimum of 500 genes is recommended for analysis (Morabito et al., 2023), we increased the number of genes in each reference dataset to between 500 and 1000. Gaussian, jittered Gaussian, and jittered vine copulas were evaluated on how well coexpression modules in reference datasets were preserved in synthetic datasets. As before, the independence copula was included as a baseline. Due to their long run times, ML Gaussian, t, and vine copulas could not be fit on datasets of this size.

Gaussian copulas show the best performance (Figure 4c), having significantly higher ${\overset{‾}{Z}}_{\text{summary}}$ statistics (paired Wilcoxon rank-sum test with FDR correction) than both jittered Gaussian ($p=5.09\times 10^{-6}$) and jittered vine copulas ($p=2.54\times 10^{-3}$). Jittered vine copulas also outperform jittered Gaussian copulas ($p=2.80\times 10^{-4}$).

### 3.4 Run time

**Figure 5:**
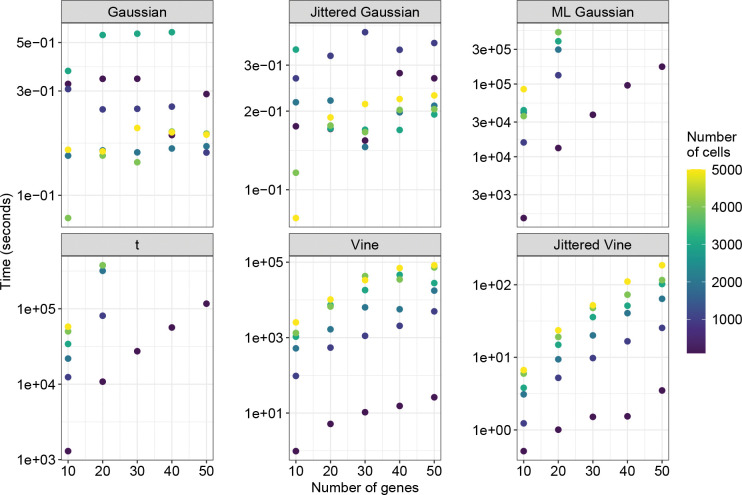
Time to fit copula models. Copula models were fit on datasets with between 100 and 5000 cells, and between 10 and 50 genes. The y-axes are on a logarithmic scale.

Finally, we evaluated the copulas on the time required to fit them. We found the run times to be quite disparate (Figure 5, Figure S3). Gaussian and jittered Gaussian copulas only require computing a sample correlation matrix. As such, they are the quickest to fit and have similar run times to each other. All other copula families require maximum likelihood estimation of some form, and therefore require substantially more time to fit. The times required to fit vine and jittered vine copulas scale roughly the same with respect to the number of cells and genes in the dataset, although vine copulas generally take several orders of magnitude longer to fit than jittered vine copulas on the same dataset. ML Gaussian and t copulas take the longest, and outside of the smallest datasets, they required more than 10 days to fit on a single core (Intel Xeon CPU @ 1.9–2.1 GHz, 50GB RAM).

## 4 Discussion

Statistical models of scRNA-seq data form the basis of many methods for analyzing scRNA-seq data. As scRNA-seq becomes an increasingly integral tool in cell-level studies, it is has become critical to develop accurate models. Statistical models of scRNA-seq data must model not only transcript counts of individual genes, but also the patterns of coexpression between genes. Copula-based modeling has emerged as a popular approach to coexpression modeling, although no studies have evaluated the performance of different copula models in doing so. In this study, we systematically evaluated six copula models on their ability to accurately and efficiently capture coexpression patterns in scRNA-seq data.

Our results suggest that there is no benefit in using a more flexible copula model as opposed to a simple Gaussian copula with a sample correlation matrix estimator. Using ML Gaussian or t copulas provides only a negligible increase in accuracy at the cost of a generally impractical amount of time required to fit them. Jittered Gaussian copulas had a markedly worse performance while offering no increase in speed. The evaluations comparing the accuracy of Gaussian and jittered vine copulas were mixed with no clear top performer. However, fitting jittered vine copulas takes several orders or magnitude longer. The only family which does show a nontrivial improvement in performance are vine copulas. However, as with the other families requiring maximum likelihood estimation using raw transcript counts, the use of vine copulas is hampered by the time required to fit them. On small datasets (< 10k cells, < 100 genes), it is feasible to fit them. On modeling tasks that require high accuracy, we would recommend vine copulas if sufficient computing resources are available. However, scRNA-seq studies now commonly sequence tens of thousands if not millions of cells, and if a large number of genes need to be modeled, vine copulas will generally be infeasible to fit. Strategies to better balance accuracy and speed for vine copulas could therefore be a possible avenue for future research.

## Supplementary Material

Supplement 1

## Figures and Tables

**Table 1:** Description of scRNA-seq studies used to generate reference datasets.

Study	Organism	Tissue	Assay(s)	Source
Bailey et al. (2024)	*Homo sapiens*	Lung	10x 5’ v1 10x 5’ v2	GSE232627
Reed et al. (2024)	*Homo sapiens*	Breast	10x 3’ v3	E-MTAB-13664
Jones et al. (2024)	*Homo sapiens*	Ovary	10x 3’ v3	GSE260685
Lukassen et al. (2020)	*Homo sapiens*	Lung	10x 3’ v2	EGAS00001004419
Orozco et al. (2020)	*Homo sapiens*	Retina	10x 3’ v3	GSE135133
Litviňuková et al. (2020)	*Homo sapiens*	Heart	10x 3’ v2 10x 3’ v3	ERP123138
King et al. (2021)	*Homo sapiens*	Tonsil	10x 5’ v1	E-MTAB-9005
James et al. (2020)	*Homo sapiens*	Colon	10x 3’ v2 10x 5’ v2	E-MTAB-8474
Gu et al. (2024)	*Mus musculus*	Retina	10x 5’ v2	sanger.ac.uk
Tritschler et al. (2022)	*Sus scrofa domesticus*	Pancreas	10x 3’ v2	GSE198623

## Data Availability

All datasets used in this study are publicly available. All scRNA-seq datasets except for Bailey24 are available in AnnData format from CZ CELLxGENE (https://cellxgene.cziscience.com/datasets). The Bailey24 dataset (Bailey et al., 2024) is available under GEO accession GSE232627. The Reed24 dataset (Reed et al., 2024) is available under EMBL accession E-MTAB-13664. The Jones24 (Jones et al., 2024) dataset is available under GEO accession GSE260685. The Lukassen20 dataset (Lukassen et al., 2020) is available under EGA accession EGAS00001004419. The Orozco20 dataset (Orozco et al., 2020) is available under GEO accession GSE135133. The Litvinukova20 dataset (Litviňuková et al., 2020) is available under ENA accession ERP123138. The King21 dataset (King et al., 2021) is available under EMBL accession E-MTAB-9005. The James20 dataset (James et al., 2020) is available under EMBL accession E-MTAB-8474. The Gu24 dataset (Gu et al., 2024) is available from https://treg-gut-niches.cellgeni.sanger.ac.uk. The Tritschler22 dataset (Tritschler et al., 2022) is available under GEO accession GSE198623. All gene sets are available from KEGG.

## Footnotes

1 Discrete covariates can be modeled by fitting copulas separately to each distinct value of the covariate.

## 7 Code availability

All code used to generate the results and figures in this manuscript is available at https://github.com/cpuritz/scrna_copula_modeling.
