# Supplementary material for: Copula modeling of gene coexpression in single-cell RNA sequencing data
Source: bioRxiv. 2025 Dec 9:2025.12.04.692380. Preprint. [Version 1] doi: 10.64898/2025.12.04.692380 (PMC12710820; doi:10.64898/2025.12.04.692380)
Supplement: Supplement 1 [file NIHPP2025.12.04.692380v1-supplement-1.pdf]

bioRxiv preprint doi: https://doi.org/10.64898/2025.12.04.692380; this version posted December 9, 2025. The copyright holder for this preprint (which was not certified by peer review) is the author/funder, who has granted bioRxiv a license to display the preprint in perpetuity. It is made available under aCC-BY 4.0 International license.

Supplementary Material

S1 Marginal distributions

We model marginal distributions empirically to avoid degrading copula parameter estimates in the case of marginal misspecification. Specifically, for a sample $\mathbf{X}_1, \ldots, \mathbf{X}_n \overset{\text{iid}}{\sim} \mathbf{X}$, we model the ith marginal distribution F_i using the estimator

$$\widehat{F}_{n,i}(x) = \frac{1}{n+1} \sum_{j=1}^{n} \mathbb{I}(X_{j,i} \leq x)\,, \tag{S1}$$

where $\mathbb{I}$ is the indicator function. Note that this estimator is $n/(n+1)$ times the typical empirical distribution function. This asymptotically negligible rescaling is used to avoid issues with evaluating quantile functions on the boundaries of the unit hypercube (Genest et al., 1995).

S2 Jittered copulas

Let $\mathbf{X}$ be a d-dimensional random variable with joint distribution function H and marginal distribution functions $F_1, \ldots, F_d$. Let $V_1, \ldots, V_d \overset{\text{iid}}{\sim} \text{Unif}(0, 1)$ be random variables that are independent of $\mathbf{X}$. Define the d-dimensional random variable $\mathbf{U}$ by

$$U_i = F_i(X_i^-) + V_i\big(F_i(X_i) - F_i(X_i^-)\big), \quad 1 \leq i \leq d\,, \tag{S2}$$

where $F_i(X_i^-)$ denotes the left-limit of F_i at X_i. The margins of $\mathbf{U}$ are uniformly distributed on the unit interval, and the joint distribution function of $\mathbf{U}$ is a copula itself (Nešlehová, 2007). This copula, which we denote as C_H^+, is a copula of H (Nešlehová, 2007). If H were continuous, then it would be the unique copula of H. In literature, C_H^+ is often referred to as the multilinear extension copula, as it can be constructed by linear interpolation on the images of the marginal distribution functions (Genest et al., 2013).

We refer to the process of constructing $\mathbf{U}$ from $\mathbf{X}$ as jittering. Other names for this technique in literature include distributional transformation (e.g. Song et al., 2024) and continuous extension (e.g. Nešlehová, 2007). Jittering before performing inference has two supposed benefits. First, it enables the use of copula parameter inference methods for continuous distributions, which tend to be much faster than those for discontinuous distributions (Panagiotelis et al., 2012). Second, it resolves the identifiability issue, since the copula of $\mathbf{U}$ is unique.

However, jittering also introduce new complications. The process of jittering introduces additional variability through the realizations of the uniform noise terms V_i. In this work, we only use a single jitter. To account for this added variability, likelihood equations could be modified to be averaged over a large number of jitters (Nikoloulopoulos, 2013). However, this will lead to longer computational times, nullifying some of benefit gained by jittering in the first place. Furthermore, the estimators obtained from jittering may not be unbiased. Nikoloulopoulos (2013) found that the maximum likelihood estimators for Gaussian copula correlation matrices obtained using jittering were biased, and often underestimated the true strength of dependence. Increasing the number of jitters reduced the variability of the estimators but did not decrease the bias.

S3 Copula inference

Let $\mathbf{X}$ be a count-valued random variable with marginal distribution functions $\mathbf{F} = (F_1, \ldots, F_d)$. Let $\mathbf{X}_1, \ldots, \mathbf{X}_n$ be independent and identically distributed samples of $\mathbf{X}$. We performed copula inference semi-parametrically using empirical estimators of the marginal distribution functions. Under mild regularity conditions, the maximum likelihood estimators of the copula parameters are consistent when using empirical estimators of count-valued marginal distribution functions (Nasri and Remillard, 2023). Here, we describe the inference procedure for each class of copulas.

bioRxiv preprint doi: https://doi.org/10.64898/2025.12.04.692380; this version posted December 9, 2025. The copyright holder for this preprint (which was not certified by peer review) is the author/funder, who has granted bioRxiv a license to display the preprint in perpetuity. It is made available under aCC-BY 4.0 International license.

S3.1 Gaussian & jittered Gaussian copulas

For the Gaussian copula, the estimator of the correlation matrix is the sample correlation matrix of the normal-transformed pseudo-observations

$$\mathbf{N}_i = \Phi^{-1}\big(\mathbf{F}(\mathbf{X}_i)\big) = \big(\Phi^{-1}(F_1(X_{i1})), \ldots, \Phi^{-1}(F_d(X_{id}))\big), \ \ i = 1, \ldots, n, \tag{S3}$$

where Φ^{-1} is the standard normal quantile function. For the jittered Gaussian copula, jittered pseudo-observations (Equation S2) are used instead of pseudo-observations.

S3.2 ML Gaussian copula

Suppose that the copula of $\mathbf{X}$ is a Gaussian copula with correlation matrix $\mathbf{R}$. The mass function of $\mathbf{X}$ is given by

$$\begin{aligned}\Pr(\mathbf{X} = \mathbf{x} \mid \mathbf{R}) &= \Pr(X_1 = x_1, \ldots, X_d = x_d \mid \mathbf{R}) \\ &= \Pr(x_1 - 1 < X_1 \le x_1, \ldots, x_n - 1 < X_n \le x_n \mid \mathbf{R}) \\ &= \int_{\Phi^{-1}(F_1(x_1-1))}^{\Phi^{-1}(F_1(x_1))} \cdots \int_{\Phi^{-1}(F_d(x_d-1))}^{\Phi^{-1}(F_d(x_d))} \phi_{\mathbf{R}}(y_1, \ldots, y_d)\, dy_1 \ldots dy_d\,,\end{aligned} \tag{S4}$$

where Φ^{-1} is the standard normal quantile function and $\phi_{\mathbf{R}}$ is the density function of a multivariate Gaussian random variable with mean $\mathbf{0}$ and covariance matrix $\mathbf{R}$ (Panagiotelis et al., 2012). The average log-likelihood of the samples $\mathbf{X}_1, \ldots, \mathbf{X}_n$ is given by

$$L_n(\mathbf{R}) = \frac{1}{n} \sum_{i=1}^{n} \log \Pr(\mathbf{X} = \mathbf{X}_i \mid \mathbf{R}). \tag{S5}$$

As there is no closed-form expression for the mass function, we compute it numerically using the method of Botev (2016) implemented in the TruncatedNormal package (Botev and Belzile, 2024). We used 1000 Monte Carlo simulations as we found this to provide a good balance between time and accuracy.

Fitting a Gaussian copula to the samples $\mathbf{X}_1, \ldots, \mathbf{X}_n$ requires estimating the correlation matrix $\mathbf{R}$. A reasonable initial estimator is $\hat{\mathbf{R}}_n$, the sample correlation matrix of $\mathbf{N}_1, \ldots, \mathbf{N}_n$, where $\mathbf{N}_i = \Phi^{-1}(\mathbf{F}(\mathbf{X}_i))$. Note however that $\hat{\mathbf{R}}_n$ is not the maximum likelihood estimator (Hernández et al., 2014). To the best of our knowledge, no closed form solution exists for the maximum likelihood estimator, and thus we need to perform numerical maximization. To do so, we need to reparametrize the likelihood function since updating elements of $\mathbf{R}$ directly during optimization may produce a matrix which is no longer a valid correlation matrix (i.e., a symmetric positive semi-definite matrix with ones on the diagonal). We can parametrize $\mathbf{R}$ as a length $d(d-1)/2$ vector using the approach of Bhat and Mondal (2021), which we describe below. Note that we will assume that $\mathbf{R}$ is positive definite. If $\mathbf{R}$ was a singular positive semi-definite matrix, the resulting distribution would be valid but degenerate.

The unique Cholesky factor of $\mathbf{R}$ can be written as

$$\mathbf{L} = \begin{bmatrix} 1 & 0 & 0 & \cdots & 0 & 0 \\ l_{2,1} & \sqrt{1 - l_{2,1}^2} & 0 & \cdots & 0 & 0 \\ l_{3,1} & l_{3,2} & \sqrt{1 - l_{3,1}^2 - l_{3,2}^2} & \cdots & 0 & 0 \\ \vdots & \vdots & \vdots & \ddots & \vdots & \vdots \\ l_{d-1,1} & l_{d-1,2} & l_{d-1,3} & \cdots & \sqrt{1 - \sum_{k=1}^{d-2} l_{d-1,k}^2} & 0 \\ l_{d,1} & l_{d,2} & l_{d,3} & \cdots & l_{d,d-1} & \sqrt{1 - \sum_{k=1}^{d-1} l_{d,k}^2} \end{bmatrix}. \tag{S6}$$

Suppose we parametrize the lower triangular elements of $\mathbf{L}$ as

$$l_{i,j} = \begin{cases} 1 & i = 1, \\ \sqrt{\prod_{k=1}^{i-1} \left(1 - h_{i,k}^2\right)} & i = j > 1, \\ h_{i,j} \sqrt{\prod_{k=1}^{j-1} \left(1 - h_{i,k}^2\right)} & i > j. \end{cases} \tag{S7}$$

bioRxiv preprint doi: https://doi.org/10.64898/2025.12.04.692380; this version posted December 9, 2025. The copyright holder for this preprint (which was not certified by peer review) is the author/funder, who has granted bioRxiv a license to display the preprint in perpetuity. It is made available under aCC-BY 4.0 International license.

This is a valid parametrization if we take $\mathbf{H} = (h_{i,j})$ to be the $d \times d$ lower triangular matrix with $h_{i,i} = 1$ and $h_{i,1} = l_{i,1}$ for all $1 \leq i \leq d$, and

$$h_{i,j} = \frac{l_{i,j}}{\sqrt{1 - \sum_{k=1}^{j-1} l_{i,k}^2}} \quad \text{for } i > j\,. \tag{S8}$$

This parametrization of $\mathbf{L}$ is useful since it ensures the diagonal elements of $\mathbf{L}$ will remain positive and real during the search process. Since the diagonal elements of $\mathbf{H}$ are all 1, we can uniquely encode $\mathbf{H}$ as a vector $\mathbf{v_H} \in (-1,1)^{d(d-1)/2}$ whose elements are the lower triangular elements of $\mathbf{H}$ in column-major order. The elements of $\mathbf{v_H}$ can then be mapped to the real line using the bijection $f : (-1,1) \to \mathbb{R}$ defined by

$$f(x) = \sigma \log\left(\frac{\arccos(x)}{\pi - \arccos(x)}\right) \tag{S9}$$

with inverse

$$f^{-1}(y) = \cos\left(\frac{\pi}{1 + \exp(-y/\sigma)}\right) \tag{S10}$$

where $\sigma > 0$ is a scale parameter. This parametrization constrains the search procedure to the space of symmetric positive-definite matrices. Numerical experiments performed by Bhat and Mondal (2021) suggest that when this parametrization of the likelihood function is maximized using the BFGS algorithm, a scale parameter of $\sigma = 1.2904$ provides a good balance between convergence time and accuracy, and thus this is the value we use. Maximization of Equation S5 was performed numerically using L-BFGS-B optimization (Byrd et al., 1995).

S3.3 t copula

Suppose that the copula of $\mathbf{X}$ is a t copula with degrees of freedom $\nu > 0$ and correlation matrix $\mathbf{R}$. The mass function of $\mathbf{X}$ is given by

$$\Pr(\mathbf{X} = \mathbf{x} \,|\, \nu, \mathbf{R}) = \int_{t_\nu^{-1}(F_1(x_1-1))}^{t_\nu^{-1}(F_1(x_1))} \cdots \int_{t_\nu^{-1}(F_d(x_d-1))}^{t_\nu^{-1}(F_d(x_d))} f_{\nu,\mathbf{R}}(y_1, \ldots, y_d)\, dy_1 \ldots dy_d\,, \tag{S11}$$

where t_ν^{-1} is the univariate t quantile function, and $f_{\nu,\mathbf{R}}$ is the density function of a multivariate t random variable with mean $\mathbf{0}$, covariance matrix $\mathbf{R}$, and degrees of freedom ν (Panagiotelis et al., 2012). The average log-likelihood of the samples $\mathbf{X}_1, \ldots, \mathbf{X}_n$ is then given by

$$L_n(\nu, \mathbf{R}) = \frac{1}{n} \sum_{i=1}^{n} \log \Pr(\mathbf{X} = \mathbf{X}_i \,|\, \nu, \mathbf{R})\,. \tag{S12}$$

As with the Gaussian copula, there is no closed-form expression for the mass function, and we compute it numerically using the method of Botev and L'Ecuyer (2015) implemented in the TruncatedNormal package (Botev and Belzile, 2024) with 1000 Monte Carlo simulations.

While maximization of Equation S12 could be performed jointly over ν and $\mathbf{R}$, we instead took a two-stage approach to reduce compute time by taking advantage of the correlation matrix maximum likelihood estimates already computed for Gaussian copulas. The two-stage maximization procedure (Zeevi and Mashal, 2002) is as follows:

1. The t copula correlation matrix $\mathbf{R}$ is estimated by the maximum likelihood estimator $\widehat{\mathbf{R}}_G$ of the correlation matrix using a Gaussian copula model.
2. The degrees of freedom ν is estimated as

$$\widehat{\nu} = \underset{\nu \in (2,\infty)}{\arg\max}\, L_n(\nu, \widehat{\mathbf{R}}_G). \tag{S13}$$

By restricting ν to be larger than 2, we avoid obtaining a distribution with an undefined covariance matrix. We solved Equation S13 numerically using L-BFGS-B optimization (Byrd et al., 1995).

bioRxiv preprint doi: https://doi.org/10.64898/2025.12.04.692380; this version posted December 9, 2025. The copyright holder for this preprint (which was not certified by peer review) is the author/funder, who has granted bioRxiv a license to display the preprint in perpetuity. It is made available under aCC-BY 4.0 International license.

S3.4 Vine & jittered vine copulas

Vine copulas were fit using the `vinecop` function in the rvinecopulib package (Nagler and Vatter, 2023). The pair copula family set included the independence copula, the Gaussian copula, and the four available one-parameter bivariate Archimedean copulas (Clayton, Gumbel, Frank, and Joe). Parameter estimation was performed using maximum likelihood estimation (`par_method = "mle"`). The Akaike information criterion (AIC) was used for family selection (`selcrit = "aic"`). For vine copulas fit directly to transcript counts, `var_types` was set to `"d"`. For jittered vine copulas, jittered pseudo-observations (Equation S2) were passed to `vinecop` instead of pseudo-observations, and `var_types` was set to `"c"`. The default settings of `vinecop` were used otherwise.

S4 Computational details

All results were generated using R 4.4.0 (R Core Team, 2024) on Northwestern's Quest high-performance computing cluster, which consists of nodes equipped with Intel Emerald Rapids Xeon Platinum 8592+ @ 1.9GHz, Intel Ice Lake Xeon Gold 6338 @ 2.0GHz, or Intel Cascade Lake Xeon Gold 6230 @ 2.10GHz processors. Gene sets were processed using igraph 2.1.4 (Csárdi and Nepusz, 2006), graphite 1.52.0 (Sales et al., 2012), org.Hs.eg.db 3.20.0 (Carlson, 2024), and AnnotationDbi 1.68.0 (Pagès et al., 2024). Multivariate elliptical distribution functions were evaluated numerically using TruncatedNormal 2.3 (Botev and Belzile, 2024). Vine copulas were fit and sampled from using rvinecopulib 0.7.2.1.0 (Nagler and Vatter, 2023). All other copulas were sampled from using copula 1.1-6 (Hofert et al., 2024). Likelihood maximization was performed using the `optim` function in the stats package (R Core Team, 2024) or the `optimParallel` function from optimParallel 1.0-2 (Gerber and Furrer, 2019). Midweight bicorrelation was computed using the `bicor` function from WGCNA 1.73 (Langfelder and Horvath, 2008). Distance correlation was computed using the `dcor` function from energy 1.7-12 (Rizzo and Szekely, 2024). Fasano-Franceschini tests were performed using fasano.franceschini.test 2.2.2 (Puritz et al., 2023). Gene coexpression module analysis was performed using hdWGCNA 0.4.07 (Morabito et al., 2023). Cohen's d was calculated using effsize 0.8.1 (Torchiano, 2020). Wilcoxon rank-sum tests were performed using coin 1.4-3 (Hothorn et al., 2006).

Figures were generated using R 4.4.3 (R Core Team, 2024) on macOS using dplyr 1.1.4 (Wickham et al., 2023), tidyr 1.3.1 (Wickham et al., 2024), ggplot2 3.5.2 (Wickham, 2016), ggsci 3.2.0 (Xiao, 2024), ggsignif 0.6.4 (Constantin and Patil, 2021), scales 1.4.0 (Wickham et al., 2025), reshape2 1.4.4 (Wickham, 2007), and patchwork 1.3.2 (Pedersen, 2024).

bioRxiv preprint doi: https://doi.org/10.64898/2025.12.04.692380; this version posted December 9, 2025. The copyright holder for this preprint (which was not certified by peer review) is the author/funder, who has granted bioRxiv a license to display the preprint in perpetuity. It is made available under aCC-BY 4.0 International license.

Study	Dataset	Genes	Cells	Description
Bailey24	bailey24-rpra04-moam	17	1030	Profibrotic monocyte-derived alveolar macrophages from donor RPRA04. Subset to top HVGs.
	bailey24-rpra09-tram	12/500	2668	Tissue-resident alveolar macrophages from donor RPRA09. Subset to top HVGs/top HVGs.
	bailey24-rpra24-tram	15/600	1672	Tissue-resident alveolar macrophages from donor RPRA24. Subset to top HVGs/top HVGs.
	bailey24-rpra29-dc2	35	602	Type II conventional dendritic cells from donor RPRA29. Subset to top HVGs.
	bailey24-rpra20-cd8t	40/500	2285	$CD8^+$ T cells from donor RPRA20. Subset to top HVGs/top HVGs.
	bailey24-rpra30-cd4t	32/825	3040	$CD4^+$ T cells from donor RPRA30. Subset to top HVGs/top HVGs.
Reed24	reed24-donor6-myoepithelial	30/20/775	1510	Basal myoepithelial cells from donor 6. Subset to top HVGs/hsa05224 gene set/top HVGs.
	reed24-donor18-myoepithelial	36/21/675	4389	Basal myoepithelial cells from donor 18. Subset to top HVGs/hsa05224 gene set/top HVGs.
	reed24-donor19-myoepithelial	45/20/725	2340	Basal myoepithelial cells from donor 19. Subset to top HVGs/hsa05224 gene set/top HVGs.
	reed24-donor26-myoepithelial	50/19	1056	Basal myoepithelial cells from donor 26. Subset to top HVGs/hsa05224 gene set.
Jones24	jones24-donor3-stromal	25/22	750	Ovarian stromal cells from donor 3. Subset to top HVGs/hsa04915 gene set.
	jones24-donor4-stromal	20/23/750	1500	Ovarian stromal cells from donor 4. Subset to top HVGs/hsa04915 gene set/top HVGs.
	jones24-donor5-stromal	15/24/900	2400	Ovarian stromal cells from donor 5. Subset to top HVGs/hsa04915 gene set/top HVGs.
Lukassen20	lukassen20-9JQK55ng-monocyte	32	1163	Monocytes from donor 9JQK55ng. Subset to top HVGs.
	lukassen20-A9LCTZng-monocyte	10	1062	Monocytes from donor A9LCTZng. Subset to top HVGs.
	lukassen20-QZY9VQng-monocyte	14	955	Monocytes from donor QZY9VQng. Subset to top HVGs.
Orozco20	orozco20-109373-mueller	20/625	2017	Müeller cells from donor 109373. Subset to top HVGs/top HVGs.
	orozco20-109829-mueller	12	1140	Müeller cells from donor 109829. Subset to top HVGs.
	orozco20-110814-mueller	42	530	Müeller cells from donor 110814. Subset to top HVGs.

bioRxiv preprint doi: https://doi.org/10.64898/2025.12.04.692380; this version posted December 9, 2025. The copyright holder for this preprint (which was not certified by peer review) is the author/funder, who has granted bioRxiv a license to display the preprint in perpetuity. It is made available under aCC-BY 4.0 International license.

Study	Dataset	Genes	Cells	Description
Orozco20	orozco20-120974-mueller	10	1427	Müeller cells from donor 120974. Subset to top HVGs.
Litvinukova20	litvinukova20-d3-myocyte	21/1000	2656	Atrial cardiac myocytes from donor D3. Cells were sequenced using 10x 3' v2. Subset to hsa04260 gene set/top HVGs.
	litvinukova20-d6-myocyte	21/525	2116	Atrial cardiac myocytes from donor D6. Cells were sequenced using 10x 3' v2. Subset to hsa04260 gene set/top HVGs.
	litvinukova20-h6-myocyte	21	1400	Atrial cardiac myocytes from donor H6. Cells were sequenced using 10x 3' v3. Subset to hsa04260 gene set.
	litvinukova20-h7-myocyte	21/575	3469	Atrial cardiac myocytes from donor H7. Cells were sequenced using 10x 3' v3. Subset to hsa04260 gene set/top HVGs.
King21	king21-BCP5-naiveB	16/950	2262	Naive B cells from donor BCP5. Subset to top HVGs/top HVGs.
	king21-BCP6-naiveB	20	1106	Naive B cells from donor BCP6. Subset to top HVGs.
	king21-BCP8-naiveB	24	477	Naive B cells from donor BCP8. Subset to top HVGs.
James20	james20-290b-memB	10	1392	Memory B cells from donor 290b. Cells in this subset were sequenced using 10x 3' v2. Subset to top HVGs.
	james20-302c-memB	36/800	1696	Memory B cells from donor 302c. Cells in this subset were sequenced using 10x 3' v2. Subset to top HVGs/top HVGs.
	james20-390c-memB	48	450	Memory B cells from donor 390c. Cells in this subset were sequenced using 10x 5' v2. Subset to top HVGs.
	james20-290b-iga	15/700	5024	IgA^{+} plasma cells from donor 290b. Cells in this subset were sequenced using 10x 3' v2. Subset to top HVGs/top HVGs.
	james20-298c-iga	26/850	4736	IgA^{+} plasma cells from donor 298c. Cells in this subset were sequenced using 10x 3' v2. Subset to top HVGstop HVGs.
Gu24	gu24-pooled-mp	10	589	Macrophages from all donors pooled. Subset to top HVGs.
	gu24-pooled-dc	46	912	Conventional dendritic cells from all donors pooled. Subset to top HVGs.
	gu24-pooled-plasma	28	1423	Plasma cells from all donors pooled. Subset to top HVGs.
Tritschler22	tritschler22-pig1-panB	10/27/650	7200	Pancreatic β-cells from pig 1. Genes were mapped to human orthologs. Subset to top HVGs/hsa04911 gene set/top HVGs.

bioRxiv preprint doi: https://doi.org/10.64898/2025.12.04.692380; this version posted December 9, 2025. The copyright holder for this preprint (which was not certified by peer review) is the author/funder, who has granted bioRxiv a license to display the preprint in perpetuity. It is made available under aCC-BY 4.0 International license.

Study	Dataset	Genes	Cells	Description
Tritschler22	tritschler22-pig2-panA	30/25	877	Pancreatic α-cells from pig 2. Genes were mapped to human orthologs. Subset to top HVGs/hsa04911 gene set.
	tritschler22-pig2-panB	20/27/875	6000	Pancreatic β-cells from pig 2. Genes were mapped to human orthologs. Subset to top HVGs/hsa04911 gene set/top HVGs.

Table S1: Description of scRNA-seq datasets used as references for simulation.

bioRxiv preprint doi: https://doi.org/10.64898/2025.12.04.692380; this version posted December 9, 2025. The copyright holder for this preprint (which was not certified by peer review) is the author/funder, who has granted bioRxiv a license to display the preprint in perpetuity. It is made available under aCC-BY 4.0 International license.

family1	family2	measure	pval	stat	padj	eff
Jittered Gaussian	Vine	dcor	7.49423e-08	4.91289	2.69792e-07	0.32265
Jittered Gaussian	Jittered Vine	dcor	1.68754e-11	5.71290	1.51879e-10	0.30620
Jittered Gaussian	Vine	pearson	1.11130e-05	4.16416	2.70316e-05	0.24387
Jittered Gaussian	Jittered Vine	pearson	9.32816e-06	4.19493	2.39867e-05	0.24081
Jittered Gaussian	ML Gaussian	kendall	9.02943e-10	5.39494	4.06325e-09	0.21006
Jittered Gaussian	ML Gaussian	dcor	8.08399e-10	5.40520	3.82926e-09	0.20630
Jittered Gaussian	t	dcor	2.24517e-07	4.76929	7.21661e-07	0.20401
Jittered Gaussian	t	kendall	1.75889e-08	5.08725	7.19547e-08	0.20078
Jittered Gaussian	ML Gaussian	spearman	3.19146e-09	5.27186	1.36777e-08	0.19285
Jittered Gaussian	t	spearman	3.85520e-08	4.99494	1.44570e-07	0.18567
Jittered Gaussian	Gaussian	kendall	9.94760e-14	5.96931	4.05009e-12	0.18539
Jittered Gaussian	ML Gaussian	pearson	3.22331e-10	5.48725	1.81311e-09	0.17121
Jittered Gaussian	t	pearson	1.65420e-07	4.81032	5.51399e-07	0.16899
Jittered Gaussian	Gaussian	spearman	3.90799e-13	5.91803	8.79297e-12	0.16673
Jittered Gaussian	Gaussian	dcor	1.35003e-13	5.95905	4.05009e-12	0.14937
Jittered Gaussian	Gaussian	pearson	7.10543e-15	6.03085	6.39488e-13	0.14723
Jittered Gaussian	Vine	kendall	3.04041e-05	3.97954	7.01633e-05	0.13656
Gaussian	Vine	dcor	2.61503e-02	2.21541	3.67739e-02	0.13202
Jittered Gaussian	Vine	spearman	5.43088e-05	3.86672	1.16376e-04	0.12262
Jittered Gaussian	ML Gaussian	bicor	3.77700e-07	4.69750	1.13310e-06	0.11700
Jittered Vine	ML Gaussian	kendall	1.92311e-08	5.07699	7.52520e-08	0.11500
Gaussian	Jittered Vine	dcor	2.22522e-01	1.23079	2.86099e-01	0.11197
Jittered Vine	ML Gaussian	spearman	1.31010e-07	4.84109	4.53497e-07	0.11045
Jittered Vine	t	kendall	7.70744e-07	4.59493	2.10203e-06	0.10636
Jittered Gaussian	Gaussian	bicor	2.28297e-11	5.69238	1.86789e-10	0.10610
Jittered Vine	t	spearman	1.73832e-06	4.47185	4.60143e-06	0.10386
Jittered Gaussian	Vine	MI	5.38876e-11	5.63084	3.46420e-10	0.09896
Jittered Gaussian	t	bicor	1.39676e-04	3.67184	2.79351e-04	0.09855
t	Vine	dcor	2.54415e-02	2.22567	3.63450e-02	0.09352
Jittered Gaussian	Jittered Vine	kendall	3.26133e-07	4.71801	1.01214e-06	0.09213
Jittered Gaussian	t	MI	1.23563e-11	5.73341	1.23563e-10	0.08474
Jittered Gaussian	Vine	bicor	1.44321e-03	3.11799	2.59778e-03	0.08438
Jittered Gaussian	ML Gaussian	MI	3.17613e-12	5.81546	5.71276e-11	0.08252
Jittered Vine	Gaussian	kendall	5.41434e-12	5.78469	6.96129e-11	0.08194
ML Gaussian	Vine	dcor	4.98621e-01	0.68719	5.54023e-01	0.08167
Jittered Vine	Gaussian	spearman	9.23208e-11	5.58982	5.53925e-10	0.07730
Jittered Gaussian	Jittered Vine	spearman	1.04856e-05	4.17441	2.62139e-05	0.07682
t	Jittered Vine	dcor	7.25823e-01	0.35898	7.67584e-01	0.07245
Gaussian	Vine	pearson	6.07569e-01	0.52308	6.58809e-01	0.06969
Jittered Vine	Gaussian	pearson	7.33469e-01	0.34872	7.67584e-01	0.06502
Jittered Gaussian	Jittered Vine	MI	1.23563e-11	5.73341	1.23563e-10	0.06299
Jittered Vine	ML Gaussian	dcor	8.34973e-01	0.21539	8.44355e-01	0.06074
t	Vine	pearson	4.12411e-01	0.83078	4.82039e-01	0.06052
Jittered Vine	ML Gaussian	bicor	3.97025e-05	3.92826	8.93305e-05	0.05837
Jittered Gaussian	Gaussian	MI	3.80851e-12	5.80520	5.71276e-11	0.05754
Jittered Gaussian	Jittered Vine	bicor	3.64891e-03	2.86158	5.97094e-03	0.05655
Jittered Vine	t	pearson	9.55433e-01	0.06154	9.55433e-01	0.05578
Vine	ML Gaussian	pearson	8.27045e-01	0.22564	8.44355e-01	0.05388

bioRxiv preprint doi: https://doi.org/10.64898/2025.12.04.692380; this version posted December 9, 2025. The copyright holder for this preprint (which was not certified by peer review) is the author/funder, who has granted bioRxiv a license to display the preprint in perpetuity. It is made available under aCC-BY 4.0 International license.

family1	family2	measure	pval	stat	padj	eff
Gaussian	ML Gaussian	dcor	4.76965e-04	3.39492	8.94310e-04	0.04940
Jittered Vine	ML Gaussian	pearson	4.24167e-01	0.81027	4.89424e-01	0.04907
Vine	ML Gaussian	kendall	8.57044e-05	3.77441	1.75304e-04	0.04875
Jittered Vine	Vine	spearman	1.96106e-03	3.03594	3.46070e-03	0.04808
Vine	ML Gaussian	spearman	6.70167e-04	3.31286	1.23092e-03	0.04604
Jittered Vine	Vine	kendall	2.27797e-03	2.99491	3.86825e-03	0.04590
Jittered Vine	Gaussian	bicor	6.24475e-07	4.62570	1.75634e-06	0.04551
Vine	t	kendall	1.56634e-02	2.40003	2.34951e-02	0.04183
Gaussian	Vine	MI	6.46175e-10	5.42571	3.42092e-09	0.04143
Jittered Vine	t	bicor	4.05445e-03	2.83081	6.51609e-03	0.04077
Vine	t	spearman	2.47490e-02	2.23593	3.59260e-02	0.04028
Gaussian	t	dcor	6.74349e-03	2.67696	1.04640e-02	0.04015
Jittered Vine	Vine	MI	7.23091e-10	5.41546	3.61545e-09	0.03452
Vine	Gaussian	kendall	5.65996e-02	1.90772	7.83687e-02	0.03150
Jittered Vine	Vine	bicor	3.16446e-03	2.90260	5.27411e-03	0.02853
Gaussian	t	MI	4.07709e-11	5.65136	3.05782e-10	0.02793
Vine	Gaussian	spearman	1.44971e-01	1.46669	1.94737e-01	0.02746
Gaussian	ML Gaussian	MI	4.69100e-11	5.64110	3.24762e-10	0.02564
Vine	ML Gaussian	bicor	1.12344e-02	2.51285	1.71373e-02	0.02483
Gaussian	ML Gaussian	spearman	3.51217e-01	0.94360	4.27156e-01	0.02458
Jittered Vine	Vine	dcor	1.62900e-01	1.40515	2.15603e-01	0.02329
Gaussian	ML Gaussian	kendall	3.40731e-01	0.96412	4.20079e-01	0.02277
Jittered Vine	t	MI	5.04453e-07	4.65647	1.46454e-06	0.02172
Jittered Vine	ML Gaussian	MI	2.13359e-04	3.57953	4.17442e-04	0.01965
t	ML Gaussian	bicor	6.01878e-05	3.84620	1.25974e-04	0.01886
Vine	Gaussian	bicor	2.77433e-01	1.09745	3.46791e-01	0.01792
ML Gaussian	Vine	MI	1.48076e-05	4.11287	3.50705e-05	0.01737
Gaussian	t	spearman	4.66798e-01	0.73847	5.25148e-01	0.01665
Gaussian	ML Gaussian	pearson	1.69227e-01	1.38463	2.20731e-01	0.01599
t	Vine	MI	4.37371e-04	3.41543	8.37518e-04	0.01504
Gaussian	t	kendall	5.79375e-01	0.56411	6.35899e-01	0.01240
t	ML Gaussian	dcor	3.72783e-01	0.90258	4.41454e-01	0.01043
t	ML Gaussian	kendall	5.15726e-05	3.87697	1.13208e-04	0.01027
Gaussian	t	pearson	6.95515e-01	0.40001	7.45194e-01	0.01011
Gaussian	ML Gaussian	bicor	2.77433e-01	1.09745	3.46791e-01	0.00971
Vine	t	bicor	4.48248e-01	0.76924	5.10663e-01	0.00936
Gaussian	t	bicor	7.95513e-01	0.26667	8.22944e-01	0.00916
t	ML Gaussian	spearman	2.03634e-03	3.02568	3.52443e-03	0.00793
t	ML Gaussian	pearson	3.67318e-01	0.91283	4.40781e-01	0.00600
Jittered Vine	Vine	pearson	7.33057e-02	1.79490	9.99623e-02	0.00512
Gaussian	Jittered Vine	MI	2.40726e-02	2.24618	3.55170e-02	0.00446
ML Gaussian	t	MI	6.74349e-03	2.67696	1.04640e-02	0.00239

Table S2: Output of paired Wilcoxon rank-sum tests on the Frobenius errors of pairwise gene association matrices. Independence copulas were excluded from testing. The columns `family1` and `family2` indicate the copula families compared. `measure` indicates the measure of association used (dcor, distance correlation; bicor, biweight midcorrelation; MI, mutual information). `pval` records the test p-value. `stat` records the test statistic. `padj` records the FDR-adjusted p-value. `eff` records the absolute value of the effect size (Cohen's d for paired samples).

bioRxiv preprint doi: https://doi.org/10.64898/2025.12.04.692380; this version posted December 9, 2025. The copyright holder for this preprint (which was not certified by peer review) is the author/funder, who has granted bioRxiv a license to display the preprint in perpetuity. It is made available under aCC-BY 4.0 International license.

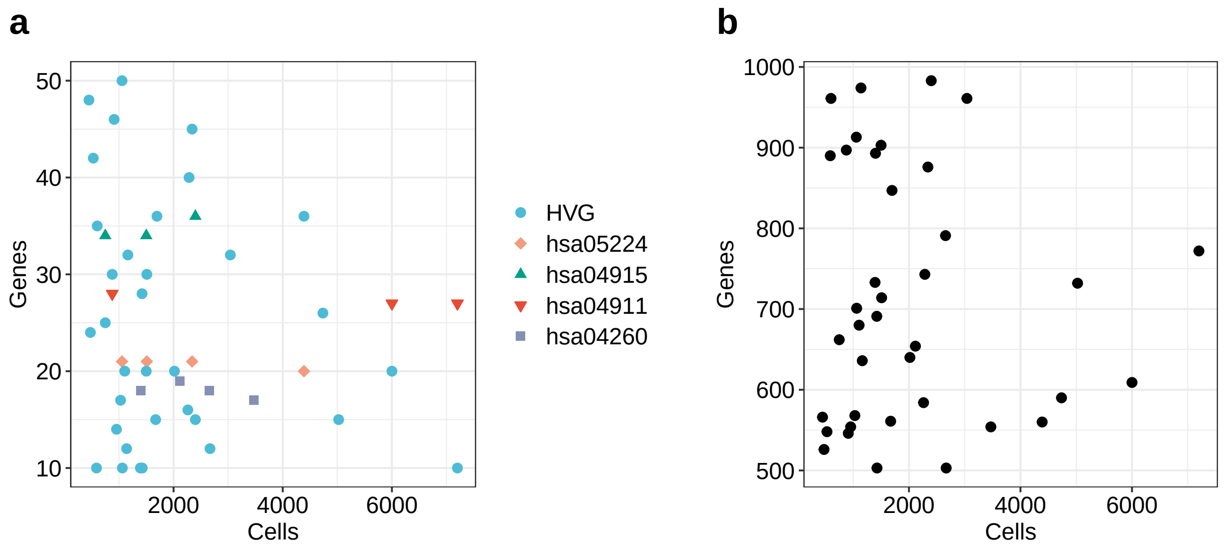

Figure S1: (a) Distribution of the number of cells and genes in reference scRNA-seq datasets used for pairwise coexpression and low-dimensional embedding evaluations. Genes for each dataset were either highly variable genes (HVG) or selected from the specified KEGG gene sets. (b) Distribution of the number of cells and genes in reference scRNA-seq datasets used for gene coexpression module evaluation.

bioRxiv preprint doi: https://doi.org/10.64898/2025.12.04.692380; this version posted December 9, 2025. The copyright holder for this preprint (which was not certified by peer review) is the author/funder, who has granted bioRxiv a license to display the preprint in perpetuity. It is made available under aCC-BY 4.0 International license.

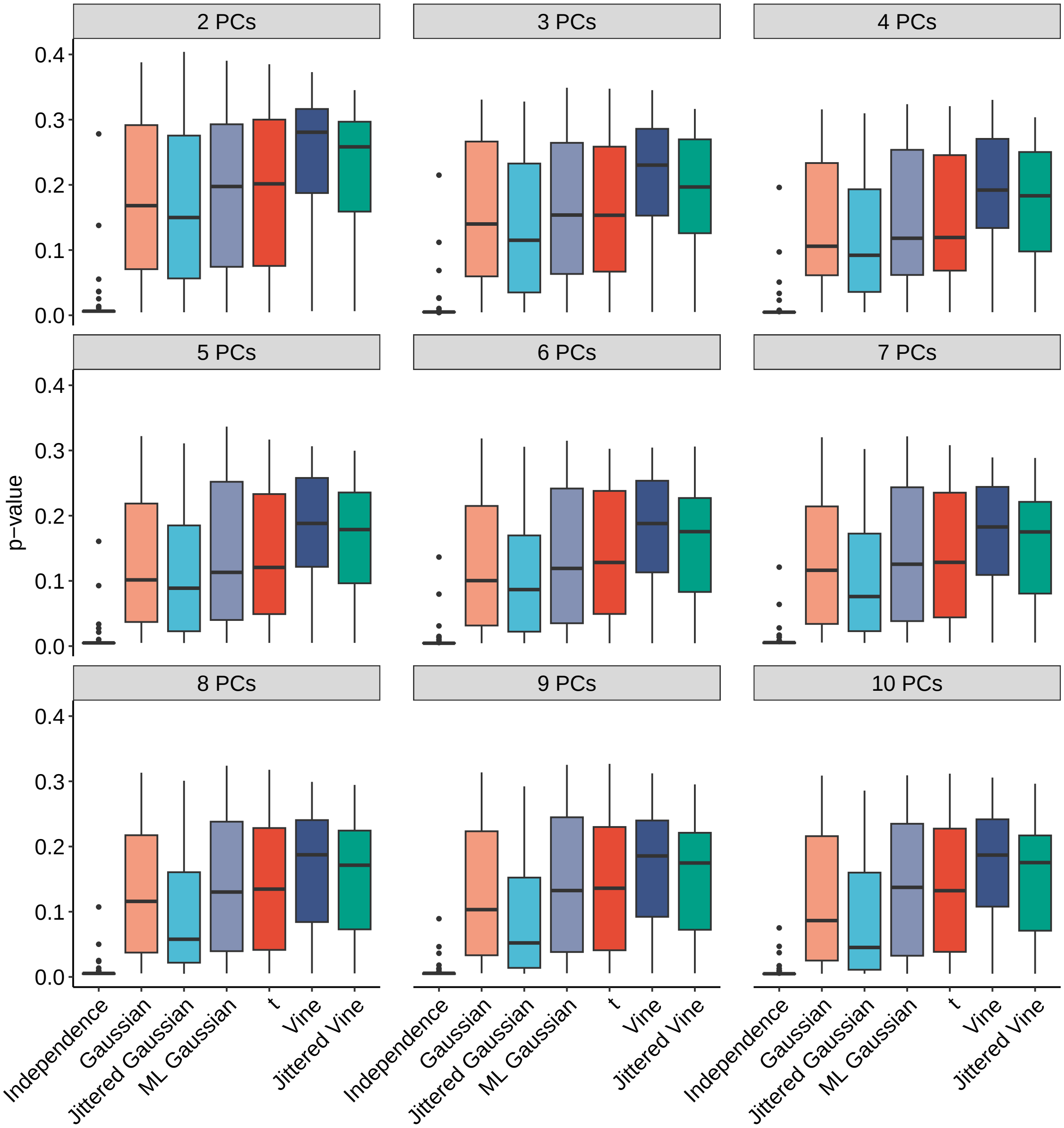

Figure S2: Evaluation of copula models using principal component (PC) embeddings with varying numbers of PCs. Each point represents the p-value from the Fasano-Franceschini test comparing the embeddings of a synthetic dataset and the corresponding reference dataset in the PC space of the reference dataset. A larger p-value indicates that the two embeddings are harder to statistically distinguish. The number of PCs retained for the embeddings is shown above each panel.

bioRxiv preprint doi: https://doi.org/10.64898/2025.12.04.692380; this version posted December 9, 2025. The copyright holder for this preprint (which was not certified by peer review) is the author/funder, who has granted bioRxiv a license to display the preprint in perpetuity. It is made available under aCC-BY 4.0 International license.

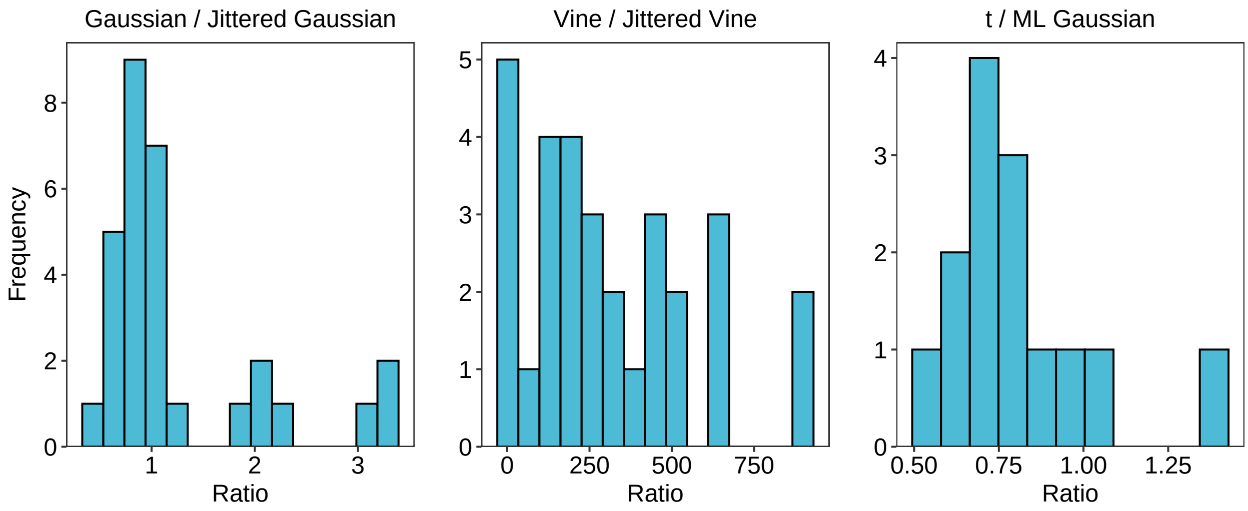

Figure S3: Ratio of times to fit different copula models on datasets of the same size. The times here are the same as reported in Figure 5. The ratio represented in each histogram is shown in the respective plot title.
